# Therapeutic Potency of Induced Pluripotent Stem-Cell-Derived Corneal Endothelial-like Cells for Corneal Endothelial Dysfunction

**DOI:** 10.3390/ijms24010701

**Published:** 2022-12-31

**Authors:** Seongjun So, Yoonkyung Park, Soon Suk Kang, Jongsuk Han, Jeong Hye Sunwoo, Whanseo Lee, Jin Kim, Eun Ah Ye, Jae Yong Kim, Hungwon Tchah, Eunju Kang, Hun Lee

**Affiliations:** 1 Department of Biomedical Science, CHA Advanced Research Institute, College of Life Science and Center for Embryo and Stem Cell Research, CHA University, Seongnam 13488, Republic of Korea; 2 Department of Ophthalmology, Asan Medical Center, University of Ulsan College of Medicine, Seoul 05505, Republic of Korea

**Keywords:** induced pluripotent stem cell, corneal endothelial cells, corneal endothelial dysfunction, cell injection, cell therapy

## Abstract

Corneal endothelial cells (CECs) do not proliferate or recover after illness or injury, resulting in decreased cell density and loss of pump/barrier function. Considering the shortage of donor cornea, it is vital to establish robust methods to generate CECs from induced pluripotent stem cells (iPSCs). We investigated the efficacy and safety of transplantation of iPSC-derived CECs into a corneal endothelial dysfunction (CED) rabbit model. iPSCs were generated from human fibroblasts. We characterized iPSCs by demonstrating the gene expression of the PSC markers *OCT4*, *SOX2*, *TRA-1-60*, and *NANOG*, teratoma formation, and differentiation into three germ layers. Differentiation of iPSCs into CECs was induced via neural crest cell (NCC) induction. CEC markers were detected using immunofluorescence and gene expression was analyzed using quantitative real-time PCR (qRT-PCR). After culturing iPSC-derived NCCs, we found the expression of zona occludens-1 (ZO-1) and Na^+^/K^+^ ATPase and a hexagonal morphology. *ATP1A1*, *COL8A1*, and *AQP1* mRNA expression was higher in iPSC-derived CECs than in iPSCs and NCCs. We performed an injection of iPSC-derived CECs into the anterior chamber of a CED rabbit model and found improved levels of corneal transparency. We also found increased numbers of ZO-1- and ATP1A1-positive cells in rabbit corneas in the iPSC-derived CEC transplantation group. Usage of the coating material vitronectin (VTN) and fasudil resulted in good levels of CEC marker expression, demonstrated with Western blotting and immunocytochemistry. Combination of the VTN coating material and fasudil, instead of FNC mixture and Y27632, afforded the best results in terms of CEC differentiation’s in vitro and in vivo efficacy. Successful transplantation of CEC-like cells into a CED animal model confirms the therapeutic efficacy of these cells, demonstrated by the restoration of corneal clarity. Our results suggest that iPSC-derived CECs can be a promising cellular resource for the treatment of CED.

## 1. Introduction

Corneal endothelial cells (CECs), embryologically originating from cranial neural crest cells (NCCs), form a single layer of hexagonal cells. CECs are responsible for barrier function between the corneal stroma and aqueous humor as they pump fluid out of the stroma to prevent edematous haze and actively transport protein and nutrients through the endothelial layer. CECs play a crucial role in controlling corneal transparency via tight-junction protein, zona occludens-1 (ZO-1), and sodium-potassium pump Na^+^/K^+^ ATPase (ATP1A1) [1,2,3,4].

CECs show no signs of regeneration in vivo because of contact inhibition at the G1 phase of the cell cycle [5,6]. Due to their lack of regeneration capability, pathological conditions such as disease and injury can cause cell loss in the corneal endothelium and consequent dysfunction of both pump and barrier. The CEC density of adult human cornea is approximately 2500 cells/mm^2^, and the cell density decreases at a rate of 0.6% per year. [5]. Corneal endothelial dystrophies and surgery-related trauma are the primary factors contributing to a decrease in endothelial cell density (ECD). If ECD decreases below 500 cells/mm^2^, the cornea deteriorates physiologically, leading to bullous keratopathy and corneal endothelial dysfunction (CED). Transplantation of a donor cornea containing healthy CECs is the only option to reverse these conditions. However, corneal graft rejection and a lack of suitable donor tissue have been primary obstacles to preventing blindness in patients with CED. [7,8]

Previously, several attempts have been made to expand human CECs from human donor corneas in vitro [9]; however, robust expansion of CECs remains a huge challenge due to the limited regenerative capacity of donor CECs [9,10]. In addition, cells from donors with older ages exhibit high heterogeneity, grow more slowly, and show senescence features compared with those from young donors [11,12]. Due to recent advances in induced pluripotent stem cell (iPSC) technology, however, it is now possible to generate an unlimited supply of CECs from iPSCs, and several groups have generated CECs from human iPSCs [13,14,15,16,17,18]. Zhao et al. generated CECs from human-fibroblast-derived iPSCs using small molecules associated with Wnt, TGF-β/BMP, and Rho-associated protein kinase (ROCK) signaling [16]. Ali et al. generated CECs from peripheral blood mononuclear cell (PBMC)-originating iPSCs through NCCs by inhibiting Wnt and TGF-β/BMP signaling (dual SMAD and Wnt inhibition) [13]. Cryopreserved and thawed corneal endothelial-like cells differentiated from iPSCs were transplanted into a monkey with corneal edema, showing improvement in the edema [17].

We developed a small-molecule-based method for induction of CECs from fibroblast-originated iPSCs and further proposed a highly efficient method to induce CECs from NCCs by replacing the coating material and ROCK inhibitor. We also evaluated the efficacy and safety of iPSC-derived CEC transplantation in a CED rabbit model.

## 2. Results

### 2.1. Characterization of iPSCs

To investigate whether iPSCs have normal PSC characteristics, we performed immunocytochemistry using PSC markers such as OCT4, SSEA4, and TRA-1-60 (Figure S1A). iPSCs expressed all the PSC marker proteins. Next, we injected iPSCs into immunosuppressed mice (NSG) to confirm the cells’ ability to differentiate. iPSCs generated teratomas containing three germ layers: endoderm, mesoderm, and ectoderm (Figure S1B). iPSCs also exhibited normal karyotyping (Figure S1C). These findings demonstrate that iPSCs have normal PSC characteristics and can be used for further studies.

### 2.2. Differentiation of iPSCs into CECs

**Figure 1:**
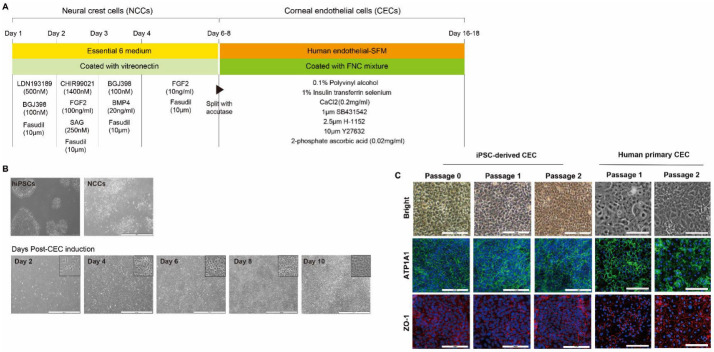
Generation of corneal endothelial cells (CECs) from induced pluripotent stem cells (iPSCs). (**A**) Protocol for differentiation of iPSCs to CECs. iPSCs were differentiated into CECs through neural crest cells for 16–18 days. (**B**) Phase-contrast microscopy at time points during CEC differentiation illustrates likely CECs at day 6, and CECs exhibiting characteristic hexagonal/polygonal morphology at day 10. Scale bars = 500 µm. (**C**) Immunocytochemistry of pump function protein Na^+^/K^+^ ATPase α1 (ATP1A1) and zona occludens-1 (ZO-1; a tight-junction protein) exhibiting hexagonal/polygonal morphology of iPSC-derived CECs. Human primary CEC was used as a control. Cell nuclei were counterstained with DAPI. Scale bars = 100 μm.

Differentiation of iPSCs into CECs via NCC was performed in vitro to determine the feasibility of iPSC-derived CEC transplantation (Figure 1A). Ten days after CEC induction from NCC, the microscopically observed phenotype of iPSC-derived CECs exhibited hexagonality and tight junctions with one another, all of which are main characteristics of CECs (Figure 1B). We measured expression of the CEC-specific proteins, ATP1A1 and ZO-1, in iPSC-derived CECs through immunocytochemistry (Figure 1C). Most of the cells expressed both ATP1A1 and ZO-1, and the expression of these markers was maintained even after two passages.

**Figure 2:**
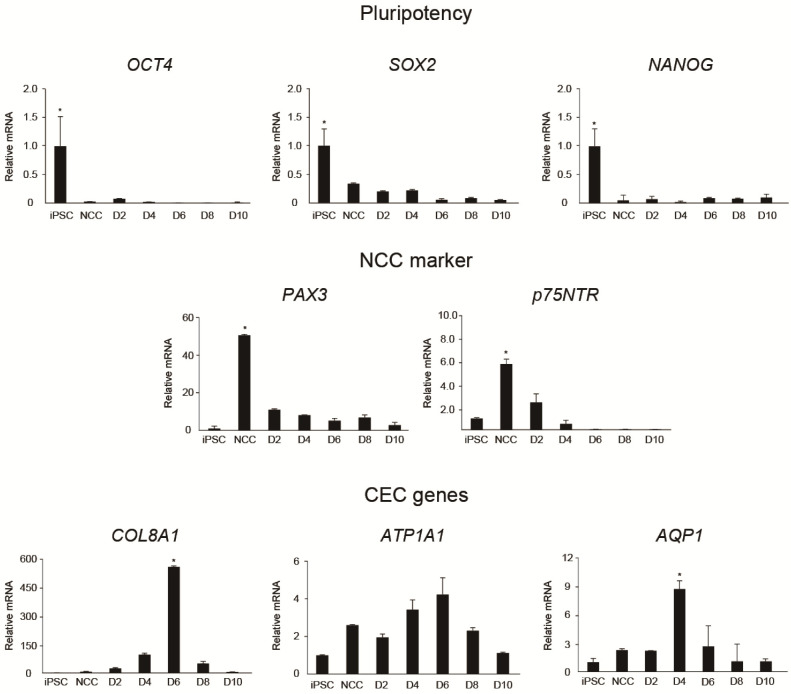
Quantitative real-time RT-PCR (qRT-PCR). qRT-PCR analysis of the expression of representative pluripotent stem cell (PSC), neural crest cell (NCC), and corneal endothelial cell (CEC) genes. NCC-related gene expression decreased as differentiation progressed, while CEC-related gene expression increased until 6 days after CEC differentiation, decreasing thereafter (* *p* < 0.05).

To determine whether differentiation to CEC was successful, we collected cells from iPSCs, NCCs, and iPSC-derived CECs on differentiation days 2, 4, 6, 8, and 10 and measured the expression of representative PSC markers (*OCT4*, *SOX2*, and *NANOG*), NCC markers (*p75NTR* and *PAX3*), and CEC markers (*ATP1A1*, *COL8A1*, and *AQP1*) with quantitative real-time PCR (qRT-PCR) (Figure 2A). PSC-related gene expression was significantly higher at the iPSC stage, whereas p75NTR and PAX3, which are NCC-related genes, were highly expressed at the NCC stage. CEC-related genes were expressed the highest on days 4–6 of CEC differentiation (*p* < 0.05). Since NCCs are able to differentiate into multi-lineages of cell type including neural lineages, we added markers for neural stem cells (nestin), immature neurons (TuJ1), and glial lineage of cells (GFAP). qRT-PCR results showed that NCCs do not differentiate into neural cells (Figure S2).

### 2.3. Stability of iPSC-Derived CECs

**Figure 3:**
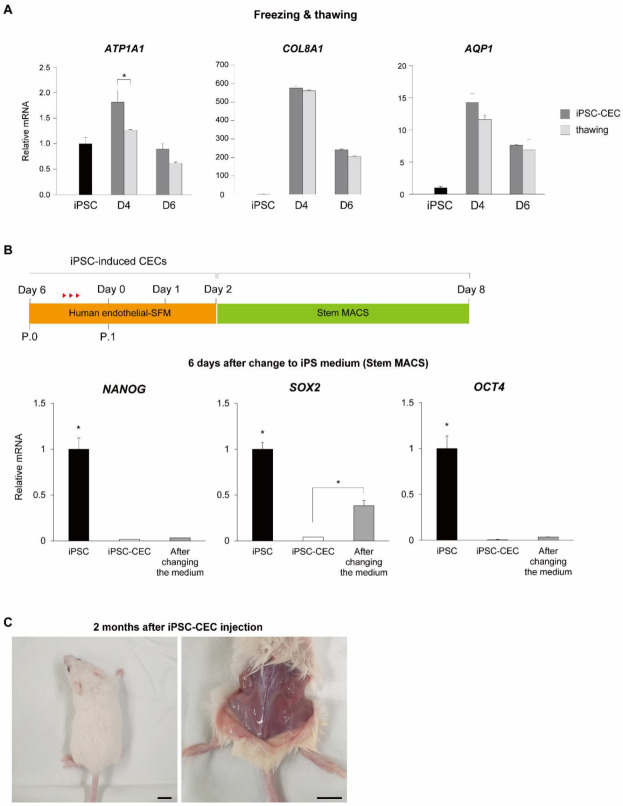
Stability of iPSC-derived CECs. (**A**) qRT-PCR analysis measuring *ATP1A1*, *COL8A1*, and *AQP1* expression relative to that of GAPDH, following freezing and thawing of iPSC-derived CECs. Expression of all three genes was maintained after freezing and thawing (* *p* < 0.05). (**B**) Experimental scheme for replacing CEC culture medium with iPSC culture medium; qRT-PCR analysis of pluripotency-related gene expression in iPSC-derived CECs 6 days after changing to iPSC culture medium. The medium was changed to iPSC culture medium 2 days after passaging of the iPSC-derived CECs, and then cultured for 6 days to analyze pluripotent gene expression. Pluripotent gene expression did not increase despite change to an iPSC culture medium, and there was no significant difference with iPSC-derived CECs (D6) except for SOX2 (* *p* < 0.05). (**C**) In vivo tumorigenicity test of iPSC-derived CECs. Tumor formation was not found even after 2 months after injection of iPSC-derived CECs into the femoral region of immunodeficient mice. Scale bars = 1 cm.

To determine whether iPSC-derived CECs maintained their characteristics after freezing and thawing, we measured the expression of CEC markers (*ATP1A1*, *COL8A1*, and *AQP1*) with qRT-PCR before and after thawing of iPSC-derived CECs. Despite freezing and thawing, there was no significant change in the expression of *ATP1A1*, *COL8A1*, and *AQP1* on days four or six of CEC differentiation, except for *ATP1A1* expression at day four of CEC differentiation (Figure 3A). Next, we cultured CECs with a Stem MACS iPS-Brew XF medium to check the reversibility of iPSC-derived CECs into iPSCs. We confirmed that the expression of pluripotent markers in iPSC-derived CECs at day six after changing to iPS medium was significantly lower than that in iPSCs (Figure 3B). Pluripotency gene expression in iPSC-derived CECs did not increase despite changing to iPSC culture medium when compared with that in iPSC-derived CECs (D6), except for SOX2. To determine whether differentiated cells exhibit tumorigenicity, we performed a teratoma formation assay using iPSC-derived CECs and compared the results with those of iPSCs (Figure 3C). We injected iPSCs (1 × 10^6^ iPSCs in 150 μL of StemMACS) and iPSC-derived CECs (1 × 10^6^ iPSC-derived CECs in 150 μL of CEC differentiation medium) into different immunosuppressed mice and observed the animals for 2 months. Teratomas were visually observed in all mice injected with iPSCs, compared to no visual observations in mice injected with iPSC-derived CECs. Mice were sacrificed to confirm teratoma formation. Thus, we determined that iPSC-derived CECs do not form teratomas in vivo.

### 2.4. Transplantation of iPSC-Derived CECs into Animals

**Figure 4:**
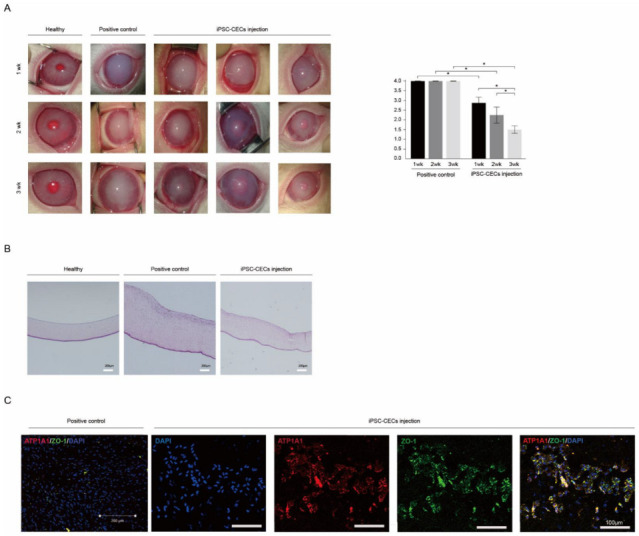
Transplantation of iPSC-derived CECs into a corneal endothelial dysfunction rabbit model. (**A**) Ocular observation and numerical data at 1, 2, and 3 weeks after transplantation of iPSC-derived CECs. Compared with the non-transplanted control, cloudy corneas were clear in the transplanted eyes after 1 week and the effect increased over time (* *p* < 0.05; n = 6). Corneal opacity was evaluated on a scale of 0–4 (0 = none; 1 = mild turbidity, iris texture evident; 2 = moderate turbidity, iris texture unclear; 3 = severe turbidity, pupil faint; and 4 = severe turbidity, pupil not visible). (**B**) The corneal thickness of the cell transplant group was observed histologically using H & E staining. The cornea of the transplanted group (**right**) is thinner compared with the non-transplanted control group (**middle**), which closely resembled the healthy cornea (**left**). Scale bars = 200 μm. (**C**) Immunohistochemistry for the detection of human CEC markers (ATP1A1 and ZO-1) in the corneas of transplanted and non-transplanted groups. ATP1A1 and ZO-1 were broadly expressed in the cornea of the transplant group, but not in the non-transplant control group. Scale bars = 200 or 100 μm.

Based on results from differentiation studies, we transplanted iPSC-derived CECs into a rabbit model of CED. This CED animal model was established in New Zealand white rabbits by stripping the Descemet’s membrane in a circular pattern. Animals were divided into groups: one transplanted with iPSC-derived CECs and another non-transplanted control group; both were observed for 3 weeks. We found that, compared with the non-transplanted control group, transplantation of iPSC-derived CECs evidently reversed cloudy corneas to relatively clear corneas within 3 weeks (Figure 4A). Moreover, assessment of corneal opacity before and after cell transplantation showed a significant improvement in corneal opacity. Compared with the non-transplanted control group, the iPSC-derived CEC injection group exhibited a lower corneal opacity score at 1, 2, and 3 weeks after transplantation (*p* < 0.05). Corneal thickness was evaluated with hematoxylin and eosin (H & E) staining; the cell transplant group showed thinner corneas than the non-transplanted control group (similar to a healthy cornea, Figure 4B). We performed an immunohistochemical analysis using cornea tissue 3 weeks after transplantation to confirm that the cells were derived from transplanted human iPSC-derived CECs as opposed to rabbit cells. An increased number of ZO-1- and ATP1A1-positive cells was found in the corneas of the rabbits transplanted with iPSC-derived CECs, when compared with the non-transplanted control group (Figure 4C). These results confirm that the in vitro differentiated CECs not only remained in the rabbit cornea for 3 weeks post-transplantation but also exhibited therapeutic effects while maintaining their histological characteristics.

### 2.5. Efficient Differentiation of iPSCs into CECs

**Figure 5:**
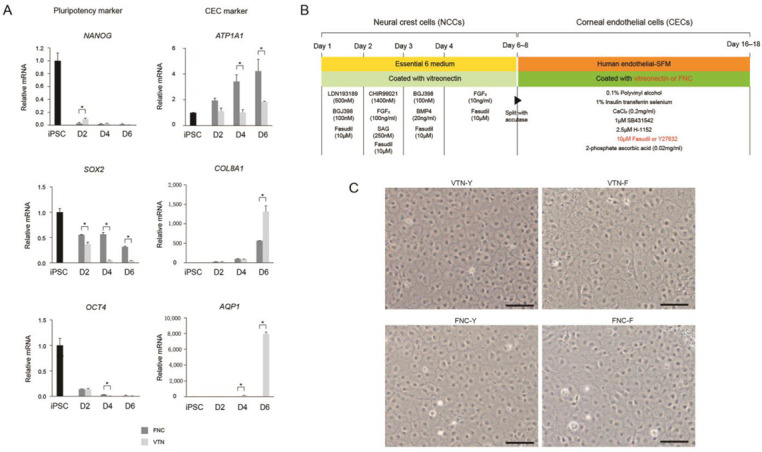
Efficient CEC differentiation by replacement of FNC coating reagent into vitronectin (VTN). (**A**) Pluripotency-related gene expression analysis of iPSC-derived CECs using VTN or FNC as coating reagents. Pluripotent gene expression of iPSC-derived CECs, differentiated using VTN, was lower compared with that of FNCs (* *p* < 0.05; n = 3). Except for *ATP1A1* expression, CEC-related gene expression was significantly higher when VTN was used (* *p* < 0.05; n = 3). (**B**) Scheme of the protocol using fasudil or Y27632 as ROCK inhibitor and VTN or FNC as coating material during CEC differentiation. (**C**) Morphology of differentiated CECs in four different groups of culture conditions: VTN-Y (Y27632), VTN-F (fasudil), FNC-Y, and FNC-F. Scale bars = 100 μm.

Next, we investigated ways to increase the efficiency of the CEC differentiation process. First, we determined the differentiation efficiency by replacing the coating material from the FNC mixture with vitronectin (VTN) for the CEC differentiation step. SOX2 expression in the FNC mixture was significantly higher than that in VTN at 2, 4, and 6 days of CEC differentiation (*p* < 0.05) (Figure 5A). COL8A1 and AQP1 expression in the VTN was significantly higher than that in the FNC mixture at 6 days of CEC differentiation (*p* < 0.05). *ATP1A1* expression in the FNC mixture was higher than that in VTN at 4 and 6 days of CEC differentiation (*p* < 0.05).

**Figure 6:**
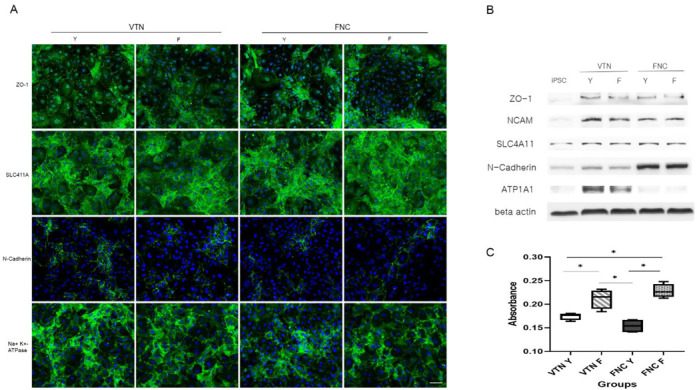
Comparison of different ROCK inhibitors and coating materials on CEC differentiation. (**A**) Immunocytochemistry using antibodies against ZO-1, SLC4A11, N-Cadherin, and ATP1A1. Scale bar = 50 μm for all the images. (**B**) Western blot using a group of CEC markers: ZO-1, NCAM, SLC4A11, N-Cadherin, and ATP1A1. (**C**) CCK-8 assay. Fasudil-treated groups had increased levels of cell viability and proliferation compared to Y27632-treated groups. (* *p* < 0.05; n = 3).

After confirming the positive effects of the VTN versus the FNC mixture, we compared the effects of Y27632 (Y) and fasudil (F) on CEC differentiation under VTN vs. FNC coating materials (Figure 5B,C). Fasudil is a ROCK inhibitor that is easier to synthesize than Y27632 and has already been approved for clinical use in some countries [19]. All four different groups of culture conditions (VTN-Y, VTN-F, FNC-Y, and FNC-F) showed quite similar morphology and the differentiation ratio we observed was >95% in all four groups (Figure 5C). Additionally, to analyze the effects of different ROCK inhibitors and coating materials, we performed immunocytochemistry and Western blot using a group of CEC markers: ZO-1, NCAM, SLC4A11, N-Cadherin, and ATP1A1 (Figure 6A,B). The VTN-coated groups showed higher levels of CEC marker expression than the FNC-coated groups, as shown by immunocytochemistry and CEC-protein expression. In addition, the CCK-8 assay showed that fasudil-treated groups had increased levels of viability and cell proliferation than Y27632-treated groups (Figure 6C). There were no noticeable differences in proliferation levels between FNC vs. VTN groups. Overall, these results indicate that NCC culture on VTN with fasudil could result in the best effects on CEC differentiation and growth compared to any other combinations.

**Figure 7:**
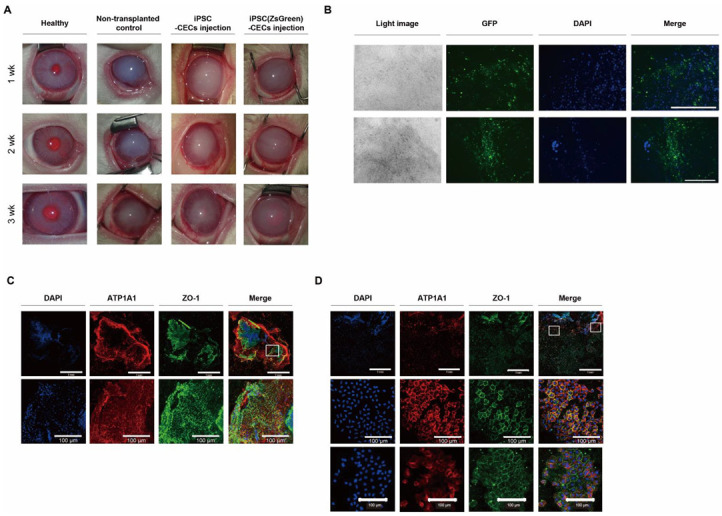
Transplantation of iPSC-induced CECs or iPSC (ZsGreen)-derived CECs produced via a modified protocol (VTN with fasudil) into a corneal endothelial dysfunction rabbit model. (**A**) Ocular observation. (**B**) Observation of transplanted iPSC (ZsGreen)-derived CECs expressing green fluorescent protein and DAPI in the iPSC-derived CEC-transplanted cornea using a fluorescence microscope. Scale bars = 400 μm. (**C**,**D**) Immunohistochemistry for the detection of human CEC markers (ATP1A1 and ZO-1) in the iPSC-derived CEC-transplanted cornea. ATP1A1 and ZO-1 were broadly expressed in the cornea of the transplant group. (**C**,**D**) show results in different corneal regions of rabbits after cell transplantation. Higher density with smaller size of transplanted CECs (**C**) and lower density with larger sized cells (**D**) were found in the cornea. The bottom rows show higher magnifications of images of the white rectangles in the top row. Scale bars = 1 mm or 100 μm.

Based on these results, we fostered CEC differentiation using green fluorescent protein (ZsGreen)-transfected iPSCs via a modified protocol (VTN with fasudil). Transplantation of iPSC (ZsGreen)-derived CECs into a CED animal model helped determine whether iPSC-derived CECs could be transplanted into the cornea region. Compared with that in the non-transplanted control group, transplantation of iPSC-derived CECs or iPSC (ZsGreen)-derived CECs reversed cloudy corneas into clear corneas within 3 weeks (Figure 7A). We observed CECs expressing green fluorescent protein in the iPSC (ZsGreen)-derived CEC transplanted cornea (Figure 7B). Furthermore, ZO-1 and ATP1A1-positive cells were found in the corneal tissue transplanted with iPSC (ZsGreen)-derived CECs (Figure 7C,D). These results confirm that the in vitro differentiated CECs from iPSCs remained in the cornea for 3 weeks post transplantation and had therapeutic effects. Finally, we evaluated the long-term effects of this transplantation of iPSC-derived CECs (Figure S3). Cloudy corneas were clear in the transplanted eyes after 3 weeks, and the therapeutic effect increased over time up to 20 weeks.

We performed immunohistochemistry on the tissue sections using antibodies against CD11b (macrophages), CD4, and CD8 (T cells) (Figure S4). We did not find any immunoreactive (IR) cells for the markers on the corneal sections of the transplant group, while a few positive cells were shown on the ciliary body, which is a vasculature-rich region. This result indicates that the host cornea could maintain immune privilege after cell transplantation and avoid corneal allograft rejection.

## 3. Discussion

Corneal transplantation using healthy CECs is the only option to treat CED. However, many issues remain to be resolved, such as a lack of donor tissue, allograft rejection, primary graft failure, and continual loss of ECD. Expanding the number of primary human CECs isolated from donor tissues also remains a challenge because of the limited regenerative capacity of CECs, due to their poor mitotic activity [20]. Therefore, there has been considerable interest in the development of in vitro expandable cell sources for endothelial regeneration.

In the present study, we describe the derivation of CECs from fibroblast-originated iPSCs, using a stepwise differentiation protocol based on small-molecule-based guidance. We demonstrated that iPSCs differentiate into NCCs, and NCCs can be converted into CECs, with hexagonal, tightly packed morphologies, expressing established markers of mature CECs. Furthermore, using in vivo experiments, we demonstrated significant corneal clarity restoration after iPSC-derived CEC transplantation in a CED animal model.

The corneal endothelium is derived from cranial NCCs in vivo [21,22]. The neural crest represents an embryonic migratory cell population that is able to distinguish itself into a variety of cell types. NCCs can differentiate into specific cell types, depending on their location, once they reach their target tissues by migrating throughout the body [23]. First, we aimed to differentiate iPSCs into NCCs. To generate iPSC-derived NCCs, we took advantage of a recently published 6-day protocol that exploits lineage-specific differentiation of human PSCs to multipotent NCCs using small-molecule compounds: LDN193189 (selective inhibitor of the BMP type I receptors ALK-2 and ALK-3), BGJ398 (potent and selective FGFR inhibitor for FGFR1/2/3), fasudil (selective ROCK inhibitor), and CHIR99021 (a potent inhibitor of GSK3, inhibiting GSK3β, GSK3α, and a Wnt activator) [19,24].

To subsequently produce CECs from iPSC-derived NCCs, we took advantage of an additional 10-day cornea endothelial induction protocol using three small-molecule compounds: SB431542, H-1152, and Y27632. During eye development, separation of the lens from the surface ectoderm is essential [25]. This separation leads to reduced signaling activity of growth factors, such as FGF2 and TGF-β, thus making it necessary to suppress the TGF-β signaling pathway with SB431542 and to remove FGF2 from the culture medium during CEC induction from NCCs. Okumura et al. demonstrated that inhibition of ROCK signaling with the small molecule Y27632 inhibited CEC apoptosis, increased CEC proliferation, and enhanced endothelial wound healing in vitro and in vivo [25,26]. H-1152, a more potent ROCK inhibitor, exhibited a larger stimulatory effect on CEC migration, proliferation, and wound healing, compared with Y27632. Thus, we used a combination of H-1152 and Y27632 to block ROCK activity more efficiently during CEC induction.

As shown, we used iPSCs to generate CECs by modifying a previously published procedure. Several groups have generated CECs from iPSCs through NCC [13,14,15,16]. Fukuta et al. established a 19-day protocol to generate CECs from human primary-fibroblast-derived iPSCs via NCC under CEC-conditioned, chemically defined media, with addition of Y27632 on the first day of CEC induction [14]. The combination of CHIR99021 and SB431542 with a minimum growth factor (insulin) efficiently induced NCCs from PSCs at a rate of 70–80%. The iPSC-derived CECs exhibited polygonal morphology, expressed the endothelial marker ZO-1, and showed increased expression of COL4A1 and COL8A1 [14]. Zhao et al. generated CECs from human foreskin-fibroblast-derived iPSCs using a small-molecule-based protocol, in which the simultaneous inhibition of TGF-β, BMP, and Wnt signaling resulted in elevated expression of early eye field transcription factors (PAX6, LHX2); an increase in Wnt signaling in the ocular niche environment is required for the formation of ocular NCCs [16]. First, they derived eye field stem cells (EFSCs) from PSCs by the addition of SB431542, LDN193189, and IWP2 (for 7 days); they then directed EFSCs toward ocular NCCs with the addition of CHIR99021 (7 days) and subsequently differentiated ocular NCCs to CECs via the addition of SB431542 and H-1152 (7 days; 21 days in total) [16]. Ali et al. generated CECs from PBMC-originated PSCs through NCCs using dual SMAD inhibitor medium containing Noggin and SB431542 for 2 days, followed by corneal medium containing Dickkopf related protein-2 (DKK-2, a potent Wnt inhibitor) for 13 days (totally 20 days) [13]. Consistent with the aforementioned studies, to generate CECs from iPSCs through NCCs, NCCs were treated with SB431542, H-1152, and Y27632 for 10 days in our study. Wagoner et al. generated CECs from adult dermal-fibroblast-derived iPSCs through NCCs. NCCs were differentiated from iPSCs by adding CHIR99021 and SB431542 for 5 to 16 days, and CECs were differentiated from NCCs by adding B27, PDGF-BB, and DKK-2 to endothelial medium for 25 to 96 days [15]. Wagoner et al. also found that the iPSC–CEC differentiation time may be reduced by restricting NCC induction to 3–8 days, demonstrating that use of early-stage NCCs can generate CECs in only 25 days [15]. Similarly, a recently published protocol was used to produce CECs directly from iPSCs by omitting NCC differentiation. These cells expressed characteristic molecules found in CECs, such as N-cadherin and PITX2 [17].

For induction of CECs from NCCs, we adopted a 10-day protocol with modifications [13]; there was no difference between expression levels of CE-associated markers at days 10 and 20 (data not shown). High expression of CE markers, and more importantly, lack of NCC marker expression, strongly encourages differentiation of NCCs into a CEC lineage. It is worth noting that we developed the 17-day protocol for the generation of CECs from iPSCs using a small-molecule-based protocol. Moreover, the combination of the VTN coating material and fasudil, instead of FNC mixture and Y27632 afforded the best results in terms of CEC differentiation’s in vitro and in vivo efficacy, showing improvements in corneal clarity.

Since fetal bovine serum (FBS) can cause an acute immune response due to any xeno-contamination after transplantation, we replaced FBS with polyvinyl alcohol (PVA). FBS used in cell culture may include contaminants which result in the presence of xeno antigens that cause graft versus host response [27]. Contamination may also result in variations between batches [28]. Based upon our results, we suggest that PVA can be a replacement for FBS.

Further studies are necessary to elucidate the functional characteristics and therapeutic potential of iPSC-derived CECs in clinical trials. Moreover, precise measurement of the population purity of a sub-lineage cell type must be determined with cell sorting based on the expression of stable and cell-type-specific markers, although an absolute marker for CECs is yet to be discovered. Functional studies should be directed to testing iPSC-derived CECs for their ability to pump water and metabolites and to survive the energy demands of ATP-dependent pump activities [29,30].

## 4. Materials and Methods

### 4.1. Ethics

This study was approved by the Institutional Review Board (IRB: 2020-1373) at Asan Medical Center. Experiments on live vertebrates were conducted in strict accordance with the relevant national and international guidelines regarding animal handling as mandated by the Institutional Animal Care and Use Committee (IACUC) of the University of Ulsan College of Medicine (Seoul, Republic of Korea). The committee reviewed and approved our animal study protocol (2019-12-184).

### 4.2. Generation of Induced Pluripotent Stem Cells

Human fibroblasts were obtained from a 7-year-old male patient and were transduced with the CytoTune-iPS Reprogramming Kit (Life Technologies, Grand Island, NY, USA) according to the manufacturer’s instructions. Fibroblasts (2 × 10^4^ cells/cm2) were plated into 24-well culture plates, coated with gelatin containing Stempro 34 medium (Life Technologies), and supplemented with L-ascorbic acid (S4641, Sigma-Aldrich, St. Louis, MO, USA), stem cell factor (Peprotech, Rocky Hill, NJ, USA), and bFGF (Peprotech) 7 days before transduction. Transduced cells were seeded onto mouse embryonic fibroblast (MEF)-coated plates 1 week after transduction. After 3 weeks, colonies were selected and expanded in stem MACS medium (Miltenyi Biotec, Bergisch Gladbach, Germany) on plates coated with vitronectin (VTN; Life Technologies) [19].

### 4.3. Teratoma Formation

Experiments for teratoma formation were performed by injection of human iPSC or iPSC-derived CECs [1 × 10^6^ iPSC or iPSC-derived CECs in 150 μL of culture medium (StemMACS or CEC differentiation medium)] into the bilateral femoral region of 7-week-old NOD-SCID Gamma mice (n = 2, NSG, The Jackson Laboratory, Bar Harbor, ME, USA) using a 1 mL syringe (Korea Vaccine Co., Seoul, Republic of Korea) [19]. After 2 months, the mice were euthanized and teratomas isolated, sectioned, and histologically characterized using H & E staining.

### 4.4. Neural Crest Cell Differentiation

For NCC differentiation, iPSCs were plated on a VTN-coated surface. After iPSCs reached approximately 30–40% confluence, the medium was switched to a neural crest induction E6 medium (Life Technologies) supplemented with 500 nM LDN19318 (Selleckchem, Houston, TX, USA), 100 nM BGJ398 (Selleckchem), and 10 μM fasudil (Adooq, Irvine, CA, USA). One day after differentiation, the medium was switched to E6 medium, supplemented with 1400 nM CHIR99021 (Peprotech), 100 ng/mL FGF2 (Peprotech), 250 nM SAG (Selleckchem), and 10 μM fasudil. The following day (day two), the medium was switched again to E6 medium supplemented with 100 nM BGJ398, 20 ng/mL BMP4 (Peprotech), and 10 μM fasudil. On day three, the medium was switched to E6 medium, supplemented with 10 ng/mL FGF2 and 10 μM fasudil. This medium was replaced each day for four days.

### 4.5. Corneal Endothelial Cell Differentiation

For CEC differentiation on day seven, iPSC-derived NCCs were detached using accutase (Life Technologies) and seeded on 35-mm plates coated with FNC mixture (United States Biological, Salem, MA, USA). The medium was switched to human CEC induction medium consisting of human endothelial-SFM supplemented with 0.1% polyvinyl alcohol (PVA; Sigma-Aldrich), 1% insulin-transferrin-selenium (Life Technologies), 0.2 mg/mL CaCl_2_ (Sigma-Aldrich), 0.02 mg/mL 2-phosphate ascorbic acid, 1 μM SB431542 (Selleckchem), 2.5 μM ROCK inhibitor H-1152 (Tocris, Abington, UK), and 10 μM Y27632 (Sigma-Aldrich) for an additional 10 days. The medium was changed every other day for those 10 days. Differentiated cells were passaged when they reached approximately 80% confluency.

To compare the effects of coating materials between the FNC mixture and VTN on CEC differentiation, iPSC-derived NCCs were split with accutase and seeded on a 35-mm plate coated with VTN instead of the FNC mixture. Quantitative real-time RT-PCR (qRT-PCR) measured concentrations of pluripotent markers (*NANOG*, *OCT4*, and *SOX2*), and CEC-associated markers (*ATP1A1*, *COL8A1*, and *AQP1*) at different time points during differentiation of iPSCs into CECs (iPSC and at 2-, 4-, and 6-days post-CEC induction). Furthermore, to compare the effects of two ROCK inhibitors (Y27632 versus fasudil) on CEC differentiation, iPSC-derived NCCs were split and seeded on 35-mm plates coated with the FNC mixture or VTN. They were differentiated with human endothelial-SFM, supplemented with 0.1% PVA, 1% insulin-transferrin-selenium, 0.2 mg/mL CaCl_2_, 0.02 mg/mL 2-phosphate ascorbic acid, 1 μM SB431542, 2.5 μM ROCK inhibitor H-1152, and fasudil instead of Y27632. CEC markers at 10 days post-CEC induction were measured via immunocytochemistry for ZO-1, SLC4A11, N-Cadherin, and ATP1A1, and via Western blot analysis for ZO-1, NCAM, SLC4A11, N-Cadherin, and ATP1A1.

### 4.6. Production of iPSC (ZsGreen)-Derived Corneal Endothelial Cells

iPSCs were transduced with Lentivirus (Vector pHIV-ZsGreen carrying the ZsGreen gene). The lentiviral particles were produced by co-transfecting the lentiviral vector and the packaging plasmids (psPAX2 and VSV-G envelope pMD2.G, addgene) into HEK293FT cells. HEK293FT cells were seeded at 90% confluency in P60 culture dish one day before transfection. On the day of transfection, pHIV-ZsGreen, psPAX2, and VSV-G envelope pMD2.G plasmids were mixed in a medium at a ratio of 2:2:1 and treated with Lipofectamine TM Transfection Reagent (Invitrogen, Carlsbad, CA, USA). The lentiviral-particle-containing supernatant was collected 2 days after transfection and concentrated using Lenti-X™ Concentrator (Takara, Kyoto, Japan) according to the manufacturer’s protocol. iPSCs were seeded in VTN-coated 12-well plates at 2 × 10^4^ cells. The next day, the lentiviral concentrate was diluted to 0.1% in culture medium and treated to iPSCs twice at one-day intervals. Expression of ZsGreen in iPSCs was confirmed by fluorescence microscopy 5 days after transduction.

### 4.7. Quantitative Real-Time RT-PCR

Total RNA from iPSCs, iPSC-derived NCCs, and iPSC-derived CECs was extracted using TRIzol reagent (Invitrogen). Complementary DNA was synthesized using a kit (Superscript III; Invitrogen) and qRT-PCR performed using the Power SYBR Green PCR Master Mix (Applied Biosystems, CA, USA) with a Step One ABI Real-Time PCR System (Applied Biosystems). Pluripotent markers (*NANOG*, *OCT4*, and *SOX2*), neural crest markers (*p75NTR* and *PAX3*), and CEC markers (*ATP1A1*, *COL8A1*, and *AQP1*) across multiple time points during differentiation of iPSCs into CECs (iPSC, NCC, and at 2, 4, 6, 8, and 10 days post-CEC induction) were measured. mRNA levels were normalized to GAPDH; primer sequences are listed in Table S1. 

### 4.8. CCK-8 Assay

Cells from each group were seeded in a 96-well culture plate at a density of 10^4^ cells/well in 100 μL of culture medium and then incubated at 37 °C for 24 h. Then, we added 10 μL of CCK-8 solution (Dojindo, Munich, Germany) to each well of the plate and incubated for 2 h in the incubator. The absorbance was measured at 450 nm using a microplate reader (Spectra Max 190, Molecular Devices, San Jose, CA, USA).

### 4.9. Immunocytochemical Staining

Human primary CECs or iPSC-derived CECs were fixed with 4% paraformaldehyde overnight at 4 °C, washed with PBS/0.05% Tween 20 (PBST) buffer, permeabilized in 0.5% Triton X-100, and blocked in PBST containing 1% bovine serum albumin (Sigma-Aldrich). Samples were incubated with primary antibodies against human anti-ZO-1 (1:250; Life Technologies), Na^+^/K^+^ ATPase α1 (1:200, Santa Cruz Biotechnology, Dallas, TX, USA), SLC4A11 (1:250, Novus, Centennial, CO, USA), and N-cadherin (1:200, Cell signaling technology, Danvers, MA, USA) overnight at 4 °C, followed by goat anti-mouse IgG Alexa Fluor 488-conjugated and donkey anti-rabbit IgG Alexa Fluor 647-conjugated secondary antibodies (1:500; Abcam, Cambridge, UK). Nuclei were counterstained with 4’, 6-diamidino-2-phenylindole (DAPI) (Sigma-Aldrich) for 10 min and fluorescence signals detected with either a fluorescence microscope (EVOS, Life Technologies) or a laser-scanning confocal microscope (Olympus, Tokyo, Japan).

### 4.10. Stability of iPSC-Derived Corneal Endothelial-like Cells

To determine whether iPSC-CECs maintained the function of CECs after freeze and thaw cycles, *ATP1A1*, *COL8A1*, and *AQP1* expression in iPSC-derived CECs was measured before and after the cycles using qRT-PCR. Additionally, to determine whether iPSC-derived CECs returned to iPSCs, iPSC-derived CECs were cultured on human endothelial-SFM for 2 days and the medium switched to Stem MACS iPS-Brew XF medium (Milteny Biotec) for 6 days. Pluripotent marker (*NANOG*, *OCT4*, and *SOX2*) expression levels were quantified using qRT-PCR 6 days after the change to iPS medium and compared with the levels in iPSCs and iPSC-derived CECs. mRNA levels were normalized to GAPDH.

### 4.11. Cell Transplantation Experiments

New Zealand white rabbits (n = 20, 1.8–2.2 kg) were placed in standard rabbit cages and housed under good environmental conditions. Room temperature was maintained at 24 °C with a 12 h light/dark cycle. After 7 days of acclimation, animals underwent the Descemet’s membrane stripping and cell injection procedures, performed by an experienced physician (HL). After intramuscular injections of 5 mg/kg tiletamine/zolazepam (Zoletil 50, Virbac Korea, Seoul, Republic of Korea) and 2 mg/kg xylazine hydrochloride (Rompun, Bayer Korea, Seoul, Republic of Korea), the anterior chamber was filled with a viscoelastic through a paracentesis incision, and the Descemet’s membrane was scored and stripped off from the posterior stroma in a circular pattern under the area of the epithelial marking using a reverse Sinskey hook. The diameter of the stripped membrane was measured for all the corneas of the experimental group and the value was 8.94 ± 0.18 mm. The viscoelastic was extensively removed by irrigating the anterior chamber using balanced salted solution (BSS). Then, a direct injection of cells was performed into the anterior chamber.

For the cell transplantation group, we injected iPSC-derived CECs (1 × 10^6^ iPSC-CECs in 150 μL of PBS supplemented with 100 μM of Y27632) into the anterior chamber through a paracentesis incision and followed for up to 3 weeks (n = 6). We also injected iPSC-CECs or iPSC (ZsGreen)-CECs, produced via a modified protocol (VTN with fasudil) (1 × 10^6^ iPSC-CECs in 150 μL of PBS supplemented with 100 μM of fasudil) into the anterior chamber and followed for up to 3 weeks (total n = 8; 4 for each iPSC-CECs). Two rabbits injected with iPSC-CECs were followed for up to 20 weeks. For the non-transplantation control group (control; n = 6), rabbits were evaluated for up to 3 weeks after Descemet’s membrane stripping. We assessed corneal opacity on a scale of 0–4 (0 = none; 1 = mild turbidity, iris texture evident; 2 = moderate turbidity, iris texture unclear; 3 = severe turbidity, pupil faint; and 4 = severe turbidity, pupil not visible) using a slit-lamp microscope, before and 3 weeks after transplantation. Whole rabbit corneas were harvested 3 weeks after cell transplantation. Transplanted iPSC (ZsGreen)-derived CECs expressing green fluorescent protein were observed using a fluorescence microscope (EVOS) with fixed excitation laser intensity (488 nm) and identical exposure times. All procedures conformed to the Association for Research in Vision and Ophthalmology (ARVO) Statement for the Use of Animals in Ophthalmic and Vision Research (ARVO Animal Policy).

### 4.12. Histological Staining and Analysis

All retrieved corneal tissues were fixed in 3.7% formaldehyde and embedded in paraffin. The paraffin blocks were sectioned into 4 μm thick slices. Paraffin sections were stained with H & E to assess corneal endothelial cell failure in Descemet’s-membrane-stripped rabbits. Three weeks following cell transplantation, H & E staining and immunohistochemistry were performed. Rabbits were sacrificed, and paraffin-embedded whole cornea tissues were incubated for 16 h at 37 °C with primary antibodies against ZO-1, Na^+^/K^+^ ATPase, CD11b (1:100, Abcam), CD4 (1:1000, Novus), and CD8 (1:1000, Abcam). The secondary antibodies included the corresponding Alexa-488, -555, or -647 fluorescent-labeled antibodies (1:1000, Abcam). Cell nuclei were counterstained with DAPI. The sections were viewed using an LSM780 confocal microscope (Carl Zeiss Meditec, Jena, Germany).

### 4.13. Western Blot Analysis

iPSC-derived CECs were harvested and lysed in RIPA lysis buffer (EPIS BIO, Sejong-si, Republic of Korea) containing phosphatase and protease inhibitors (Sigma-Aldrich) according to the manufacturer’s protocol. Cell lysates were separated on 8% SDS–polyacrylamide gels (SDS–PAGE) and subsequently transferred to PVDF membranes (Sigma-Aldrich) using a Trans-Blot-Cell (Bio-Rad Laboratories Inc., Richmond, CA, USA) as previously described. Non-specific protein binding was blocked with 3% BSA (Sigma-Aldrich) in PBST. Membranes were incubated with antibodies against ZO-1 antibodies (1:1000), NCAM (1:200, R & D systems, Minneapolis, MN, USA), SLC4A11 (1:250, Novus), N-cadherin (1:200, Cell signaling technology), and β-actin (1:1000; Cell signaling technology) overnight. Membranes were washed and incubated with an anti-mouse secondary antibody conjugated to horseradish peroxidase (GeneTex, Hsinchu City, Taiwan) at a 1:10,000 dilution. Detection was performed with the Gel DocTM XR+ (Bio-Rad Laboratories Inc.) according to the manufacturer’s instructions.

### 4.14. Statistical Analysis

All experiments were performed on at least three independent biological samples, and data are presented as means ± standard deviation (SD). Statistical analysis was performed with GraphPad Prism 6.0 software (GraphPad Software, San Diego, CA, USA). Comparisons of three or more data sets were performed using a one-way or two-way ANOVA followed by Bonferroni’s multiple comparison tests. Two-group comparisons were made using two-tailed Student’s *t*-tests. A value of *p* < 0.05 was considered statistically significant.

## 5. Conclusions

We established a method for the generation of CECs through the NCC lineage using iPSCs as a donor source, with an overall goal to maximize the availability of these cells for treating CED. Moreover, we suggest that a different combination of coating material and ROCK inhibitor may yield CECs from iPSCs more efficiently. Finally, results of the in vivo experiments indicate that iPSC-derived CECs can rescue corneal endothelial failure by improving corneal clarity. Differentiated CECs from iPSCs are stable and have similar gene and protein expression to CECs with efficacy of iPSC-derived CEC injection in vivo, making them promising cellular resources for the treatment of CED.

## Data Availability

Not applicable.

## Supplementary Materials

The following supporting information can be downloaded at: https://www.mdpi.com/article/10.3390/ijms24010701/s1, Figure S1: Characterization of iPSCs.; Figure S2: Quantitative real time RT-PCR data of neural cell lineage marker.; Figure S3: Long-term effects of transplantation of iPSC-derived CECs produced via a modified protocol (VTN with fasudil) into a corneal endothelial dysfunction rabbit model.; Figure S4: Histological examination for the presence of immune cells. Table S1: Primer list.
