# Supplementary material for: Therapeutic Potency of Induced Pluripotent Stem-Cell-Derived Corneal Endothelial-like Cells for Corneal Endothelial Dysfunction
Source: Int J Mol Sci. 2022 Dec 31;24(1):701. doi: 10.3390/ijms24010701 (PMC9821383; doi:10.3390/ijms24010701)

Table S1. Primer list

Gene	Primer sequence
OCT4	F: 5’-GAC AGG GGG AGG GGA GGA GCT AGG -3’
	R: 5’-CTT CCC TCC AAC CAG TTG CCC CAA A -3’
SOX2	F: 5’- GGA GAG AGA AAG AAA GGG AGA GA -3’
	R: 5’- GAG AGA GGC AAA CTG GAA TCA G -3’
NANOG	F: 5’- TGA ACC TCA GCT ACA AAC AG -3’
	R: 5’- TGG TGG TAG GAA GAG TAA AG -3’
PAX3	F: 5’- CCA CAA GAT CGT GGA GAT GG -3’
	R: 5’- ACC GCG TCC TTG AGT AAT TT -3’
P75NTR	F: 5’- TGG CCT ACA TAG CCT TCA AGA -3’
	R: 5’- GAG ATG CCA CTG TCG CTG T -3’
AQP1	F: 5’-ACC TCC TGG CTA TTG ACT ACA-3’
	R: 5’- TAT TTG GGC TTC ATC T-3’
COL8A1	F: 5’-CCT GGG TCA GCA AGT ACC TC-3’
	R: 5’-TTG TTC CCC TCG TAA ACT GG-3’
ATP1A1	F: 5’-ACA GAC TTG AGC CGG GGA TTA-3’
	R: 5’-TCC ATT CAG GAG TAG TGG GAC-3’
NESTIN	F: 5’ -TGGAGGCAAAGAGGG TTCAG-3’
	R: 5’ -TCGGAGAACTCTGTCCCCAG-3’
TUJ1	F: 5'- GTA TCC CGA CCG CAT CAT -3'
	R: 5'- TCT CAT CCG TGT TCT CCA -3'
GFAP	F: 5′-AGA AGC TCC AGG ATG AAA CC-3′
	R: 5′-AGC GAC TCA ATC TTC CTC TC-3′
GAPDH	F: 5′-GCC TCA AGA TCA TCA GCA ATG-3′
	R: 5′-TGG TCA TGA GTC CTT CCA CGA-3′

‘

Figure S1. Characterization of iPSCs. (A) Immunofluorescence analysis of OCT4, SSEA-4, and TRA-1-60 expression in induced pluripotent stem cells (iPSCs). iPSCs expressed all pluripotency marker proteins. Scale bars = 100 μm. (B) Ectodermal, mesodermal, and endodermal tissues associated with teratoma formation in iPSCs. In iPSC-derived teratomas, all three germ layers were well-formed. Scale bars = 200 μm. (C) Illumina Veriseq-based copy number variation (CNV) analysis on iPSCs: all 23 autosomes and both sex chromosomes exhibited normal CNV phenotypes.

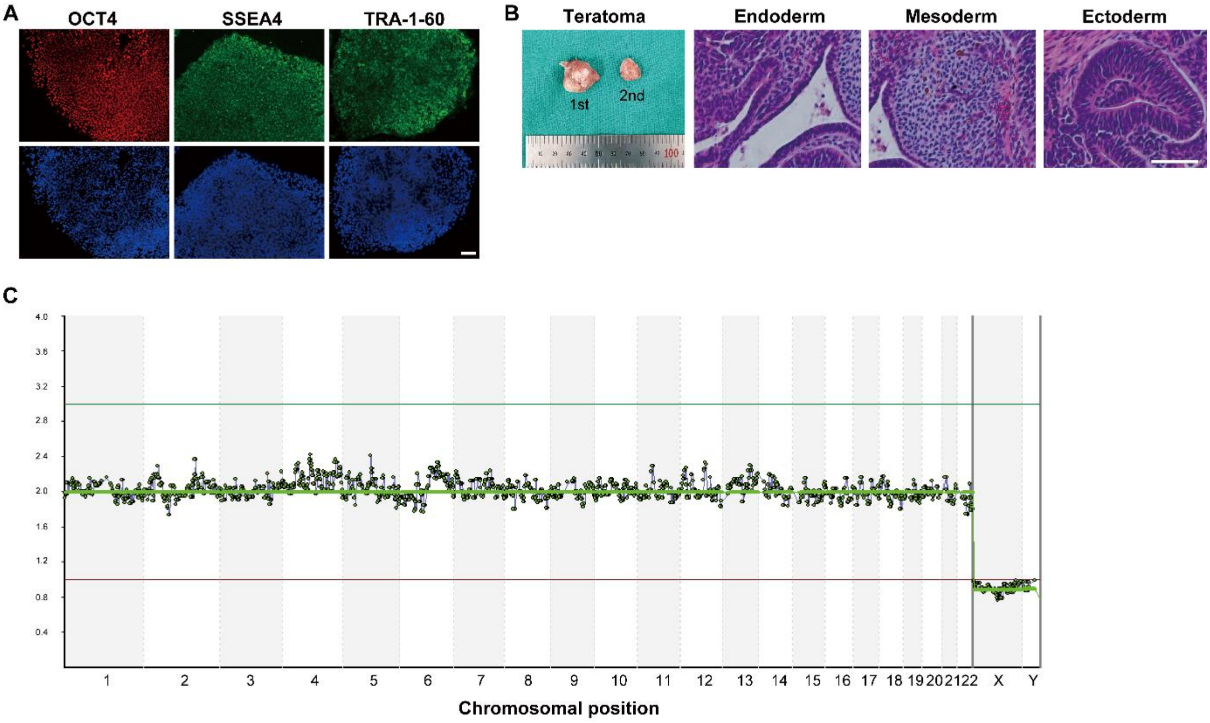

Figure S2. Quantitative real time RT-PCR data of neural cell lineage marker. The results showed that NCC did not differentiate into neural cell types (Nestin for neural stem cells, TuJ1 for immature neurons, GFAP for glial cells). No detectable levels of GFAP gene expression were found and it was not displayed in the graph.

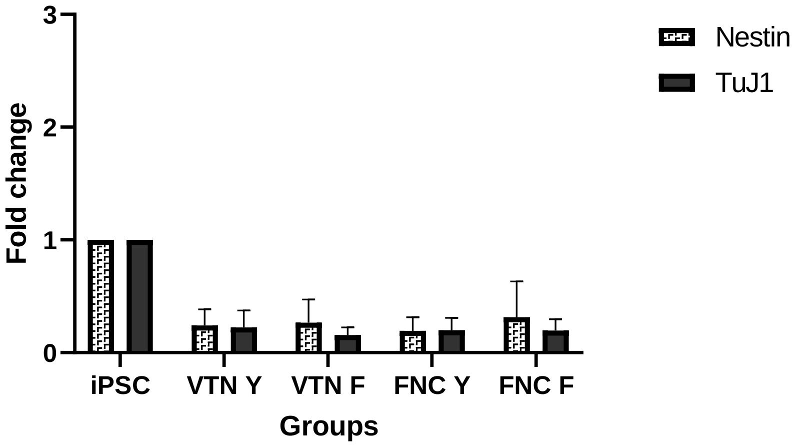

Figure S3. Long-term effects of transplantation of iPSC-derived CECs produced via a modified protocol (VTN with fasudil) into a corneal endothelial dysfunction rabbit model. Cloudy corneas cleared in the transplanted eyes after 3 weeks, and the effect increased over time.

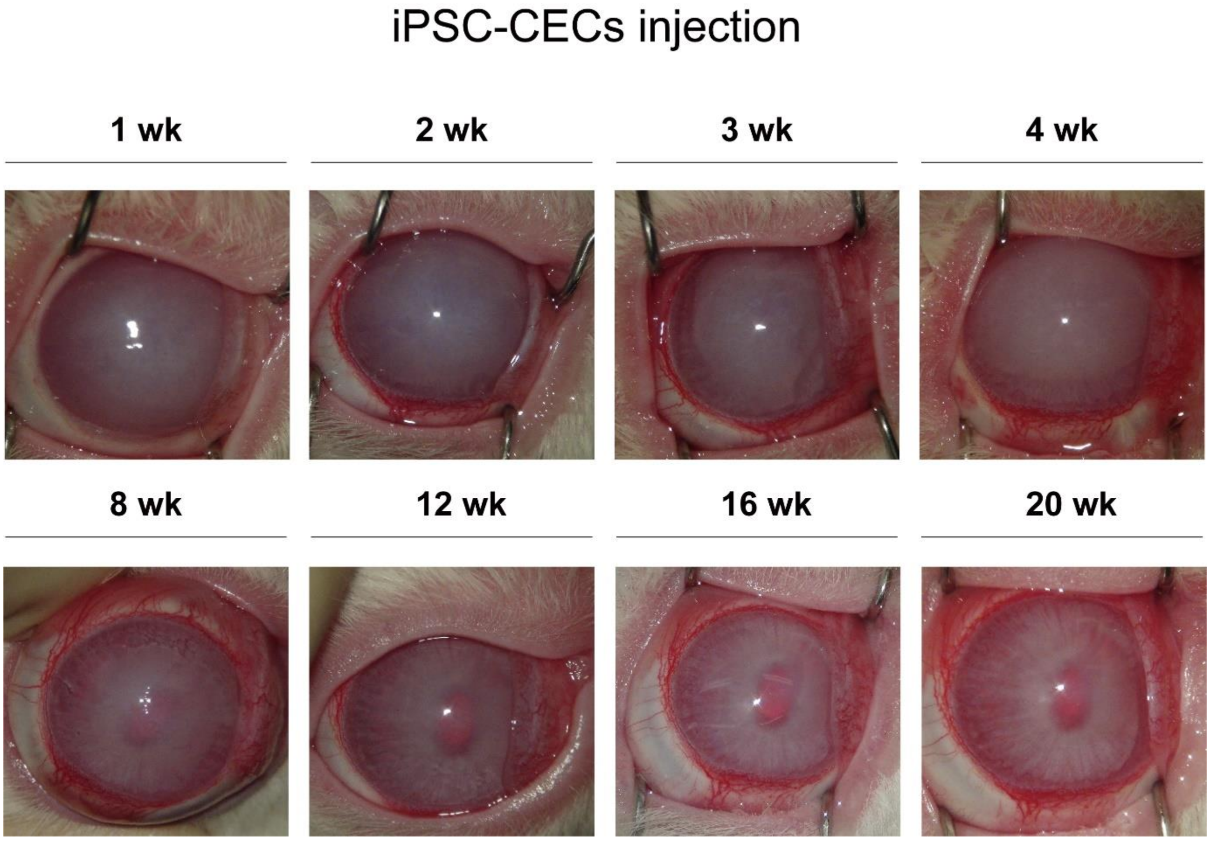

Figure S4. Histological examination for the presence of immune cells. Immunohistochemistry was performed using antibodies against CD11b (macrophages), CD4 and CD8 (T cells). There were no immunoreactive (IR) cells for any of the markers on the corneas with transplanted cells, while a few positive cells were found on the ciliary body which is a vasculature-rich region. Scale bars = 100 µm.

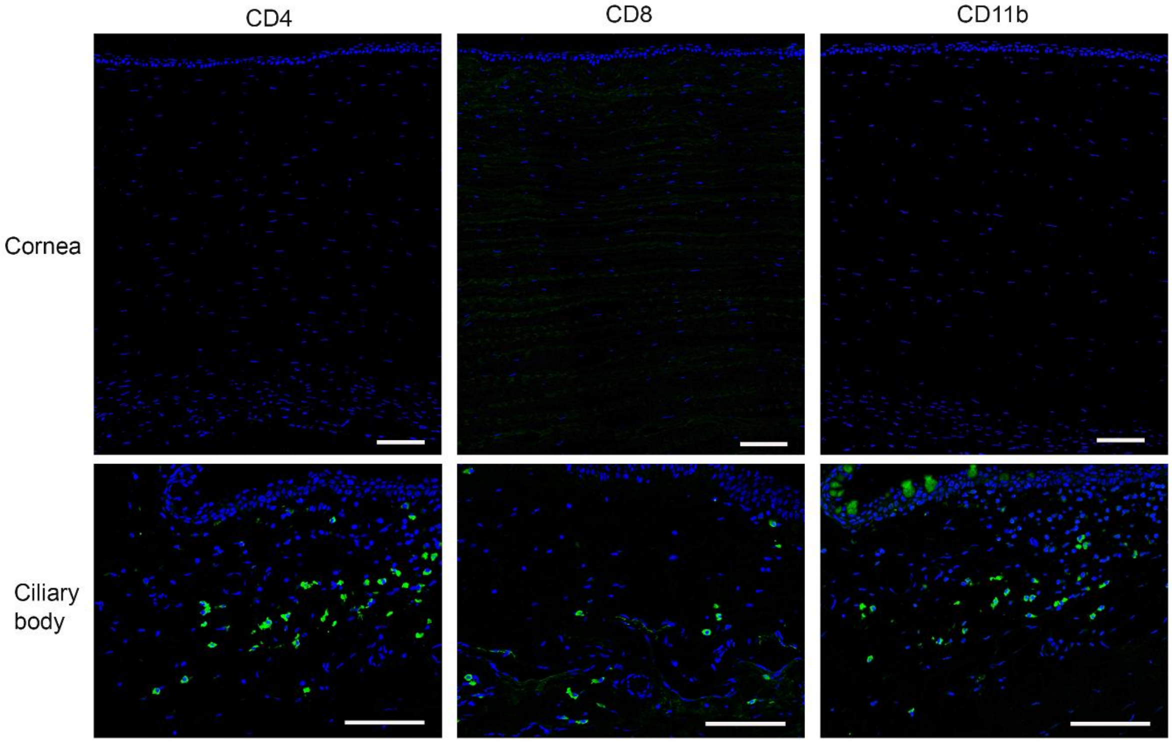

Supplement: Supplementary file 1 [file ijms-24-00701-s001.zip › ijms-1823943-supplementary.pdf]
